# Optimizing Antenna Positioning for Enhanced Wireless Coverage: A Genetic Algorithm Approach

**DOI:** 10.3390/s24072165

**Published:** 2024-03-28

**Authors:** Francisco Calles-Esteban, Alvaro Antonio Olmedo, Carlos J. Hellín, Adrián Valledor, Josefa Gómez, Abdelhamid Tayebi

**Affiliations:** Computer Science Department, Universidad de Alcalá, 28801 Alcala de Henares, Spain

**Keywords:** antenna positioning, wireless communications, genetic algorithms, propagation losses, optimization

## Abstract

The precise placement of antennas is essential to ensure effective coverage, service quality, and network capacity in wireless communications, particularly given the exponential growth of mobile connectivity. The antenna positioning problem (APP) has evolved from theoretical approaches to practical solutions employing advanced algorithms, such as evolutionary algorithms. This study focuses on developing innovative web tools harnessing genetic algorithms to optimize antenna positioning, starting from propagation loss calculations. To achieve this, seven empirical models were reviewed and integrated into an antenna positioning web tool. Results demonstrate that, with minimal configuration and careful model selection, a detailed analysis of antenna positioning in any area is feasible. The tool was developed using Java 17 and TypeScript 5.1.6, utilizing the JMetal framework to apply genetic algorithms, and features a React-based web interface facilitating application integration. For future research, consideration is given to implementing a server capable of analyzing the environment based on specific area selection, thereby enhancing the precision and objectivity of antenna positioning analysis.

## 1. Introduction

In the current landscape of wireless communications, characterized by an unprecedented increase in mobile connectivity and user density, antenna positioning emerges as a critical factor for the development of both efficient and reliable networks. The strategy for locating these antennas directly impacts fundamental aspects such as signal coverage, service quality, and the overall network capacity, making the optimization of their positioning imperative [1,2,3]. This optimization constitutes a multidimensional challenge that encompasses technical, economic, and regulatory aspects, stimulating the search for innovative solutions that merge signal propagation theory with advances in computational intelligence [1,2,4].

In this sense, the antenna positioning problem (APP) is identified as a critical challenge in the planning and management of cellular networks, having a decisive impact on network quality. This problem, which is fundamental at all stages of the lifecycle of mobile networks from 2G to 4G and LTE, encompasses frequency allocation, mobility management, and the optimization of antenna location and number to ensure maximum coverage [1,2,4,5,6,7].

Recognized as an NP-hard binary optimization problem, the APP necessitates sophisticated approaches for its solution. Through a variety of mathematical formulations and models, such as those introduced by Reininger and Calégari et al. [8,9], the goal is to achieve optimal coverage of specific geographical areas with the smallest possible number of antennas. These models account for signal path loss, influenced by physical and environmental factors, and the impact of obstacles on signal transmission and reception [5,10].

The evolution of methodologies for solving the APP has been significant, ranging from techniques based on graph theory to the implementation of genetic algorithms and the exploitation of artificial intelligence through deep learning and reinforcement learning. As mentioned in [2], pioneering work related to the APP was conducted within the framework of the STORMS project, part of a European research initiative under the ACTS Program (Advanced Communications, Technologies, and Services). The main goal of this project was the development of sophisticated tools for the design and optimization of communication networks, which included the use of specific optimization algorithms for antenna localization [11]. The contribution of [11] focused on the mathematical formulation and modeling of the APP, highlighting the inherent complexity in solving this problem. In this context, Calegari et al. stood out by publishing several works on the APP, particularly one that presents a detailed mathematical formulation aimed at solving radioelectric coverage over a surface through a biologically inspired genetic algorithm [9]. These research efforts laid the groundwork for the development of automated tools aimed at overcoming the challenge of antenna positioning.

Since their inception, a broad array of algorithms and applications has been developed and refined to tackle the challenge of antenna positioning. Notable advancements include the development of the greedy algorithm [12] and the implementation of sophisticated distributed genetic algorithms [13], evolving towards more complex multiobjective approaches [14]. Furthermore, innovative variants of genetic algorithms inspired by quantum mechanics (QIGA) have been explored, proving their validity through real case studies and detailed statistical analysis [2]. Bayesian optimization, compared with other methodologies, has been tested in applied contexts, showing promising results in studies such as those presented in [7]. Research like [15] has experimented with radio maps for air-to-ground signal propagation, applying advanced models such as channel tomography and ray tracing. Studies such as [16] have utilized common reference symbol measurements in LTE environments to assess communication quality. On another note, [17] introduced a software-defined radio (SDR) architecture capable of processing cellular signals from both CDMA and LTE, marking a milestone in cellular-assisted navigation. In [18], a comprehensive review of the APP was conducted, and the ViehFinder system was introduced. Additionally, [19] stands out for its contribution to the state of the art in this field, providing a detailed overview of the methods and solvers employed. This documentary work is particularly relevant for its clear presentation of the techniques and tools used.

In recent years, artificial intelligence has played a pivotal role in the evolution of optimization techniques and network design. A clear example of this is the work presented in [4], which demonstrates how deep reinforcement learning, an advanced technique in reinforcement learning, yields promising results surpassing traditional metaheuristic approaches such as genetic algorithms, simulated annealing, and particle swarm optimization. Similarly, [20] applies deep deterministic policy gradient (DDPG), a variant of reinforcement learning, to optimize random search, showcasing significant advancements in problem-solving efficiency. Moreover, innovative methodologies are being introduced, such as the MENA-TOA method described in [5], which integrates the Cramer–Rao bound estimator and multilateration algorithm, enhanced through the Levenberg–Marquardt method and singular value decomposition [6].

The innovations presented in these works represent a significant advancement in overcoming the challenges associated with network design and optimization, paving the way for advanced solutions in precise antenna positioning. The review of these advancements not only highlights the inherent technical complexity but also underscores the importance of adopting interdisciplinary approaches that merge signal propagation theory with the latest developments in computational intelligence. This encompasses the development of ubiquitous applications capable of working with real geographical data, aligning with the vision foreseen by Chamaret et al. [11], who were pioneers in propelling the development of automated tools to tackle the antenna positioning challenge.

As discussed in [19], to date, no standard model has been identified that can comprehensively address the APP. In fact, finding a multiobjective solution to this challenge is highly complex, leading to the formulation of a wide range of methods over time in an attempt to resolve it. Consequently, various single-objective solutions have emerged, each developed from specific contexts and limitations considered by researchers. Therefore, many of the applications developed currently are based on specific models or specific proposals designed to address the APP, as highlighted earlier.

In this context, research has focused on the development of a web-based tool aimed at enhancing signal coverage and quality in various environments through the utilization of genetic algorithms for antenna location optimization. Versions of this tool have been previously introduced by the authors, as evidenced in some of their works [21,22]. Particularly, in [23], a version solving the APP using genetic algorithms and integrating propagation losses through the Hata model [24] from a propagation server was presented. In this paper, additional models for calculating propagation losses were reviewed and integrated into the web tool to be utilized in the antenna positioning optimization. The reviewed models are Hata, Cost-231-Hata, FSPL, Egli, ECC-33, Ericsson, and Sui. The review of the models focused on the type of environment in which they are implemented (urban, suburban, or rural), operating frequency, and heights of base station (BS) and mobile station (MS) antennas, as well as the distance between them.

Upon integration into the web tool, it was demonstrated that the selection and configuration of models for calculating propagation losses enable a comprehensive analysis of antenna positioning in any area using the genetic algorithm. Additionally, it was noted that the web tool was developed using Java 17 and TypeScript 5.1.6, leveraging the JMetal framework to optimize genetic algorithms, and it features a React-based web interface that streamlines application integration.

The structure of the remainder of the document is organized as follows: Section 2 presents the materials and methods. Section 3 focuses on the results and their analysis. A brief discussion is also presented. Section 4 covers the conclusions and future work.

## 2. Materials and Methods

### 2.1. Propagation Models

The optimization of antenna positioning plays a crucial role in the efficient design and deployment of wireless networks. Determining radio propagation characteristics for a specific terrain is a key consideration in this process [10], and for this purpose, radio propagation models are useful [25]. The selection and application of these models depend on the radio propagation environment, which can be divided into rural, suburban, and urban areas [10]. Each environment presents unique characteristics due to various obstacles that affect radio wave propagation, such as reflection, refraction, diffraction, scattering, or blocking [10,25,26]. All these elements must be considered in the path loss model, which represents the decrease in signal power as it propagates from the transmitter to the receiver [10,27].

There are several propagation models that can be deterministic or empirical, and even a combination of both [10,26,28]. Of these, empirical methods are the most commonly used due to their simplicity and applicability to any experimental area [10,25,26,27]. These empirical models are strictly derived from field measurements and rely on path loss prediction [25,26,29]. In this study, seven of these models were examined: Cost-231-Hata [24,25,28,30,31,32], FSPL [27,30], Egli [26,30], ECC-33 [26,31,33], Ericsson [31,33], and Sui [31,32,34], in addition to the Hata model [24,28,29].

This models review focuses on crucial aspects for calculating propagation losses, addressing factors such as the specific implementation environment, which may vary among urban, suburban, or rural settings. Additionally, antenna operating frequency is taken into account, as this variable can significantly influence the propagation characteristics of electromagnetic waves. The heights of both base station (BS) and mobile station (MS) antennas are also considered, as the relative elevation between these antennas can impact signal quality and coverage. Lastly, the distance between antennas is evaluated, as it plays a crucial role in signal attenuation and, consequently, in wireless communication quality. These factors are thoroughly considered in the method review to ensure precise and suitable selection in antenna positioning optimization.

Table 1 displays the results of the review, detailing the seven evaluated models in the first column. The second column indicates whether the environment is influential, with responses Yes or No. In the affirmative case, users can select among urban, suburban, or rural environments in the application. Columns 3 through 6 present the range of operating frequencies, as well as the height of the BS and MS, along with the distance between them.

The outcome displayed in Table 1 was ultimately integrated into the web application, representing an enhancement for both propagation loss calculation and antenna positioning optimization through genetic algorithms in various propagation environments via the tool.

### 2.2. Genetic Algorithm for Antenna Positioning

**Figure 1:**
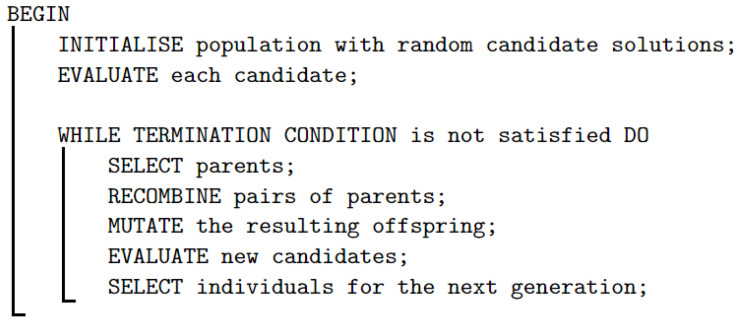
Pseudocode of the genetic algorithm.

Various approaches to address the APP, highlighting the effectiveness of evolutionary algorithms, are presented in Section 1. These algorithms, grounded in competition for limited resources and principles of natural selection, generate random candidate solutions that are evaluated and selected for reproduction through crossover and mutation operators, as shown in Figure 1.

In this study, antenna positioning optimizers are employed, utilizing propagation loss calculation as the foundation. Unlike previous research, which only considered the propagation losses of a single model, as evidenced in [23], this study incorporates the propagation losses from any of the seven models reviewed in Section 2.1, enabling the genetic algorithm to optimize the antenna position in a specific area.

The fundamental idea is to minimize the function $f\left(x\right)=\frac{1}{n}\sum_{i=1}^{n}x_{i}$, such that the position of the antenna that provides Min[f(x)] will be the optimal position of the antenna on the map, ensuring maximum propagation with the fewest possible losses [23]. The values indicated by $x_{i}$ represent the propagation losses in each point of the grid. It is important to note that the selected area is divided into tiles for the potential location of the antenna. By averaging the losses per location, an optimal position with the fewest average losses can be found. This selection is made using a genetic algorithm, which evaluates the “fitness” of each tile as a viable solution, storing this information in a cache memory. Here, the geographical coordinates (latitude and longitude) generate a unique key for each location among all possible tiles, allowing us to check if the data on propagation loss for that specific point already exists in the cache. This way, the need to perform redundant calculations of propagation losses, a process that could be very demanding from a computational standpoint, is avoided. This process is well suited for positioning one or multiple antennas, but it is always necessary to place a first antenna in a selected scenario to obtain the initial set of propagation losses, from which the genetic algorithm will start the optimization. To optimize the position of a new antenna, the calculation of the set of propagation losses will be associated with all antennas previously positioned optimally.

## 3. Results and Discussion

In this section, a brief overview of the web application and its capabilities is presented, along with some of the outcomes that can be achieved by analyzing a specific area to provide coverage for communication services.

### 3.1. Web Application for Propagation Loss Analysis and Antenna Positioning Optimization

**Figure 2:**
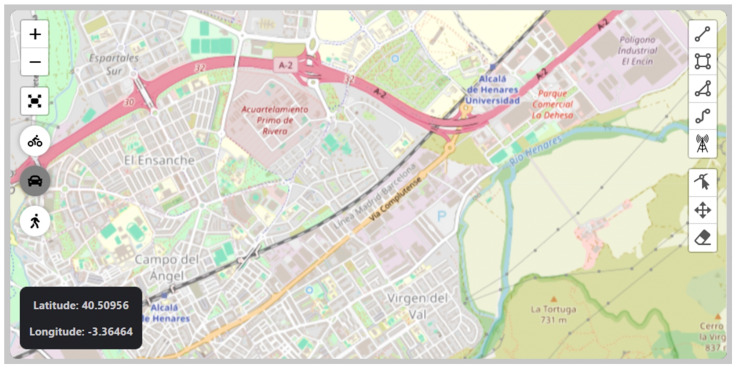
Main application window.

The web application was intuitively designed for propagation loss analysis and antenna positioning optimization. It features a main interface where various tools (icons) can be found for selecting and configuring specific areas, as depicted in Figure 2. This main interface resembles a map, allowing users to navigate and locate the area for analysis. Additionally, if users have the coordinates of the area, the tool provides methods for manually inputting these data, thereby enhancing result accuracy.

**Figure 3:**
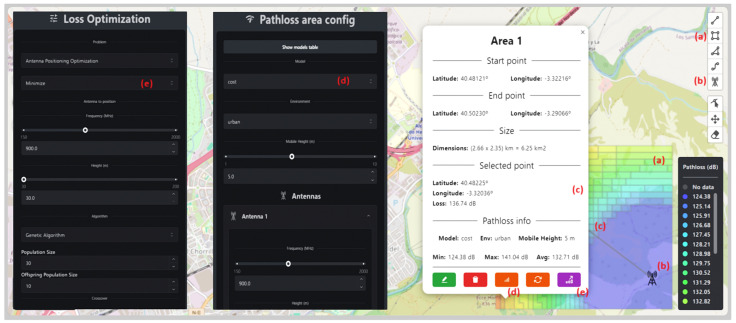
Screenshot of the graphical user interface. (a) Rectangle tool to select an area. (b) Option to add antennas. (c) Modal window that provides functionalities to calculate propagation losses. (d) The button in the middle of the bottom part of the modal window opens a new form to configure the simulation parameters. (e) The button in the right of the bottom part of the modal window opens a new form to configure the optimization parameters.

Figure 3 displays various functionalities available within the graphical user interface. The area under analysis can be determined using the rectangle drawing tool. However, the target area can also be determined by drawing polylines, polygons, or even routes defined by a start point and an end point. Additional functionalities enable the movement, editing, or deletion of elements. Selecting an area using the rectangle tool, as indicated by (a), triggers the option to add antennas, marked by (b). Clicking on the selected area launches a modal window, depicted by (c), equipping the user with tools to calculate propagation losses. Actions are executed via buttons in this modal window, which also open specific forms for configuring parameters related to propagation loss and optimization, as pointed out by (d) and (e), respectively.

Once the area is selected, users have the capability to conduct propagation loss analysis using any of the models integrated into the tool, as detailed in Table 1 of Section 2.1. It is important to emphasize the extensive capabilities offered by this tool, as any selected and configured model will send a request to the propagation server, which will calculate the losses associated with the defined area. With these data, antenna positioning optimization can be initiated using the genetic algorithm, as described in Section 2.2.

**Figure 4:**
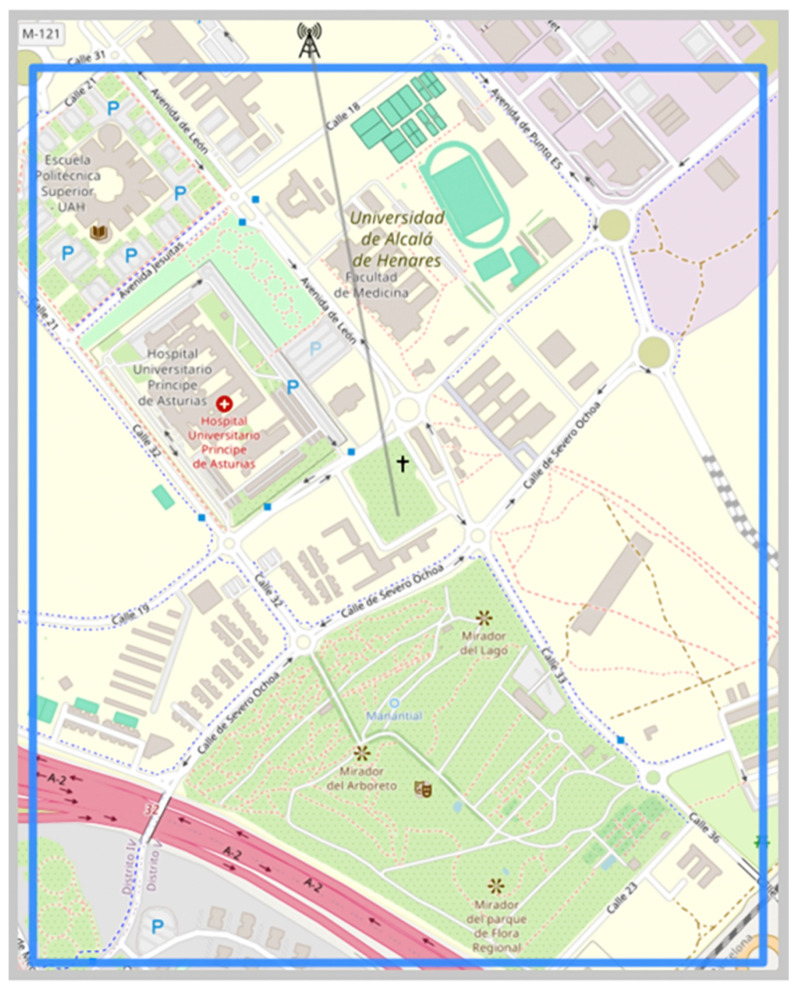
Selected area.

To comprehend the possibilities in propagation loss analysis and antenna positioning optimization, an area in Alcalá de Henares, Spain, was selected, as shown in Figure 4. To initiate the analysis and simulation of antenna positioning optimization, at least one transmitting antenna is required to calculate propagation losses. Figure 4 illustrates that the antenna is positioned outside the selected area. However, this initial placement can be either inside or outside the designated zone. The location will be determined by the type of analysis intended to optimize communication service coverage in the target area. In this scenario, the base transmitting antenna was configured to operate at a frequency of 1000 MHz with a height of 30 m. Subsequently, the antenna to be positioned, also a transmitter or BS, needs to be configured identically to the first one. An optimization of its position will be conducted using a genetic algorithm based on propagation loss calculations. Additionally, the receiving antennas or MS must be configured, with only the height fixed at 2 m. It is essential to note that, in the experiment, a height of 5 m was established for the mobile station in the ECC model, in accordance with the values in Table 1. In the Sui model, the table presents limitations in frequency, so the transmitting antennas were configured at 1900 MHz.

Table 2 presents the results of the average loss calculations for the selected area under various environments. It is important to note that the FSPL and Egli models are environment-independent, as reflected in column 2, demonstrating consistency with the review conducted in Table 1. Additionally, Table 2 highlights the optimal coordinates where the web application, through the use of a genetic algorithm, efficiently locates the BS transmitter for each of the assumed environments.

**Figure 5:**
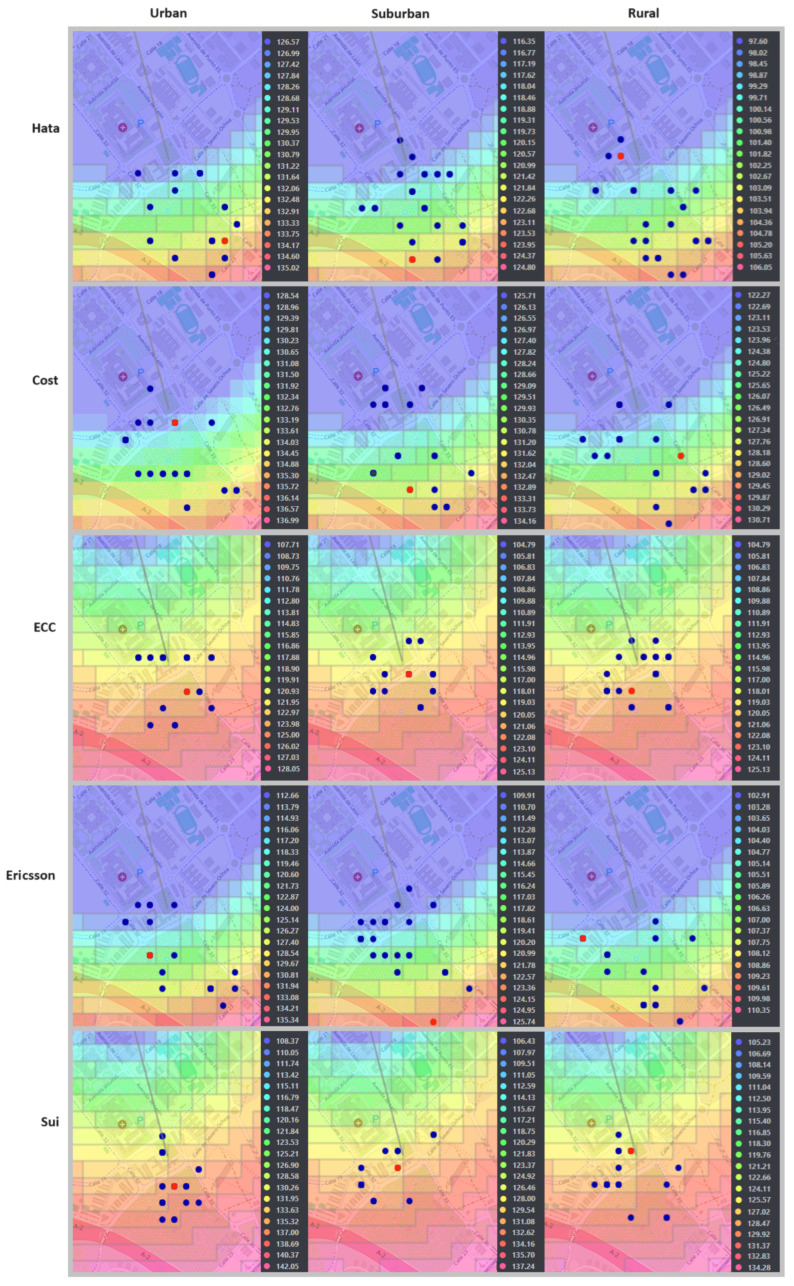
Optimization of antenna positioning for environment-dependent models.

**Figure 6:**
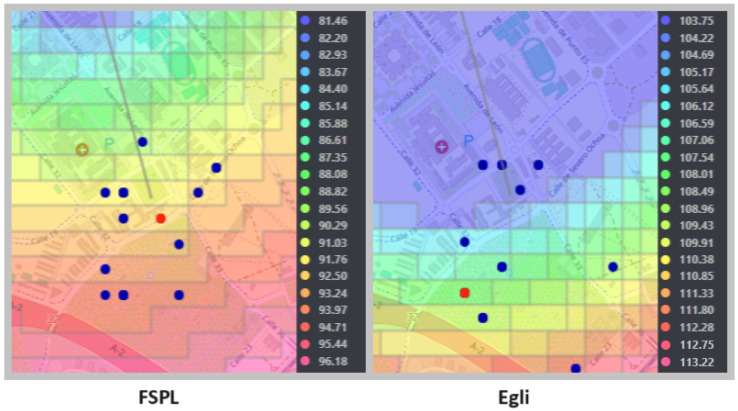
Optimization of antenna positioning for environment-independent models.

**Figure 7:**
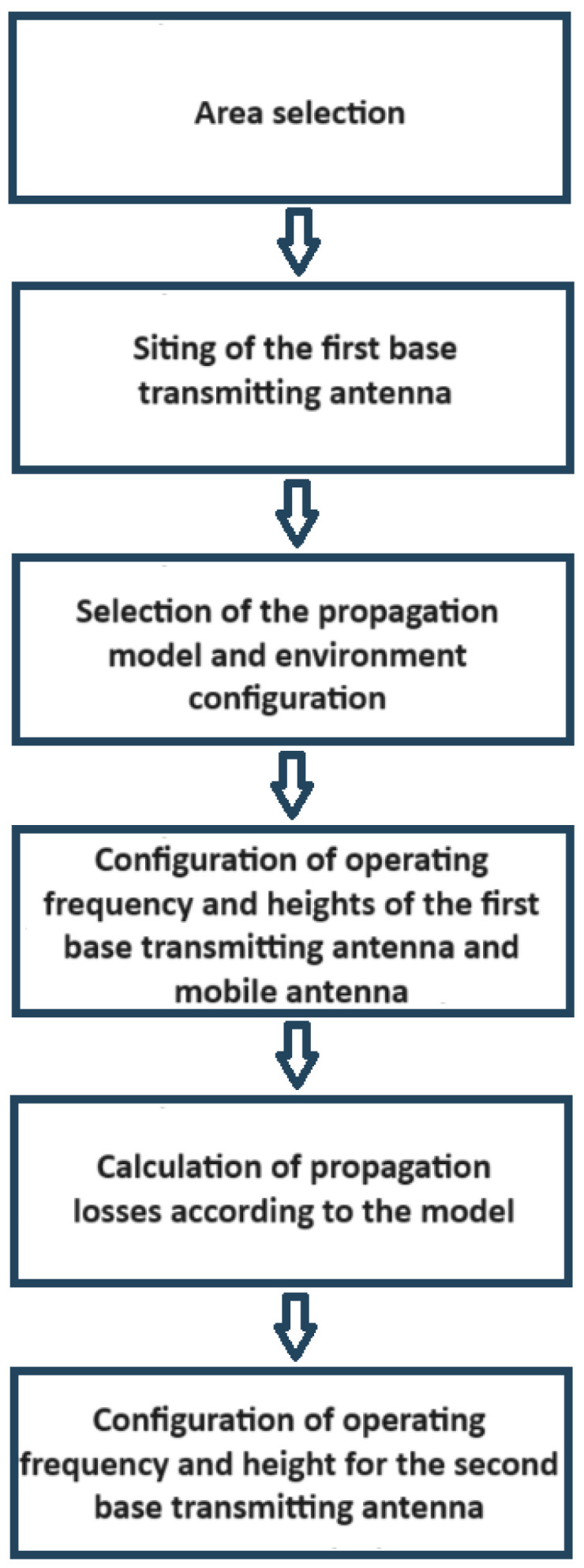
Process that determines the optimal coordinates for placing of a second base transmitting antenna.

In Figure 5 and Figure 6, the optimal positions of the transmitting antenna for both environment-dependent and independent models are clearly shown, marked with a red dot. The points evaluated by the genetic algorithm, considered as potential candidate locations, are highlighted in blue. Additionally, the coverage provided by the antennas is illustrated through an area dyed in violet, while propagation losses are indicated by a range of colors that extends from light blue to pink. For a deeper understanding of the antenna positioning optimization process, a detailed diagram is included in Figure 7, thus providing an intuitive visual representation through the web application.

Although this initial experiment was conducted using all the models outlined in Table 1 for the specified area in Figure 4, where environments (urban, suburban, or rural) are assumed, it is important to note that not all models presented in Table 1 are applicable to every area. In fact, each model has its own limitations regarding operating frequency, heights of base and mobile antennas, the distance between these antennas, and, of course, the surrounding environment. Therefore, the user conducting the analysis should be familiar with the type of environment and understand how the selected model operates.

Concluding this subsection, the results indicate that the inclusion of more models makes this web application a versatile tool, with broad capabilities for efficiently analyzing a variety of environments. As observed, currently, the application does not identify the type of environment (urban, suburban, or rural); this setting must be performed by the user. However, in the future, a server capable of assessing the environment based on the selection of a specific area could be implemented. This would enable the tool to be more precise and objective in analyzing a given area, thereby improving the accuracy of propagation loss calculations and antenna positioning optimization.

### 3.2. Optimization for Antenna Placement

In Section 3.1, a general overview is provided on how the analysis of a selected area can be conducted using any of the models integrated into the web application, along with the resulting propagation losses and their utilization in antenna positioning optimization. The web application is capable of determining the coordinates for antenna placement. Additionally, it is emphasized that not all methods can be applied universally, as they have limitations outlined and summarized in Table 1. Furthermore, emphasis is placed on the user’s need to understand both the environment under analysis and the empirical model chosen.

**Figure 8:**
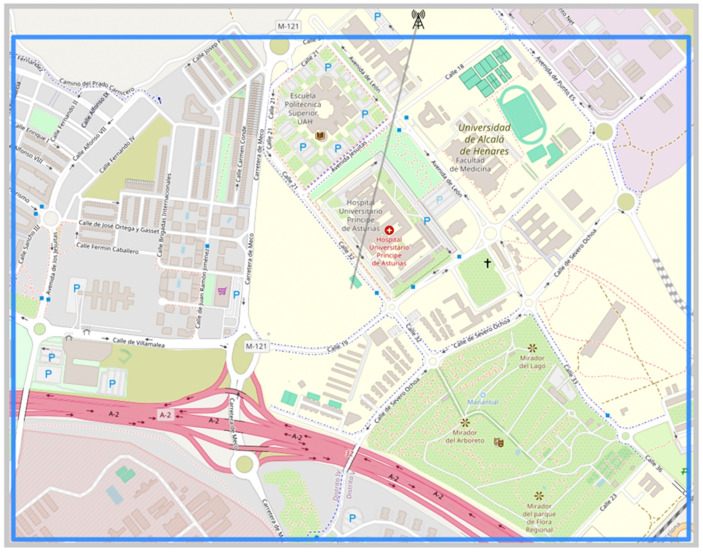
Selected area for placement of a second base transmitting antenna.

In this section, a more detailed analysis will be conducted on the calculation of propagation losses and their application in antenna positioning. To achieve this, a larger area located in Alcalá de Henares, Spain, was selected. The selected area corresponds to a suburban zone with a lower population density compared to an urban environment. In Figure 8, the selected area can be observed, characterized by few medium-rise buildings primarily dedicated to universities, hospitals, and sports facilities, as well as ample outdoor parking lots and wide, tree-lined streets with generous spacing between them [10].

Considering that the area in question is 2.60 square kilometers and consists of suburban terrain, the Cost model is selected for calculating propagation losses. The same configuration described earlier is established for the first BS transmitting antenna, with an operating frequency of 1000 MHz and a height of 30 m, as well as for the second antenna to be positioned. Moreover, the configuration of the receiving antennas or MS is defined with a fixed height of 2 m. With these parameters established, the set of propagation losses is calculated, followed by the calculation of the average, as indicated in Section 2.2.

**Figure 9:**
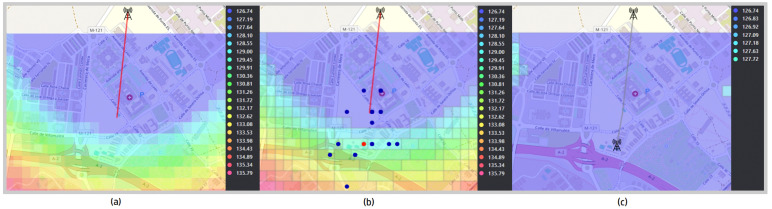
Propagation losses and coverage when placing a second antenna.

The average losses for the selected area are 131 dB, with coverage provided by a first transmitting antenna, as depicted in Figure 9a. It should be reiterated that the placement of the first antenna adheres to the analytical criteria determined by the user, as outlined in Section 3.1. The optimal coordinates for placing the second antenna are at a latitude of 40.506 degrees and a longitude of −3.349 degrees, marked by a red dot in Figure 9b. When positioning the second antenna, the average losses decrease to 127 dB. It is important to note that losses are depicted using a color gradient, ranging from light blue to pink. Figure 9c clearly illustrates the expanded coverage for both antennas.

### 3.3. Optimization for Positioning Multiple Antennas

**Figure 10:**
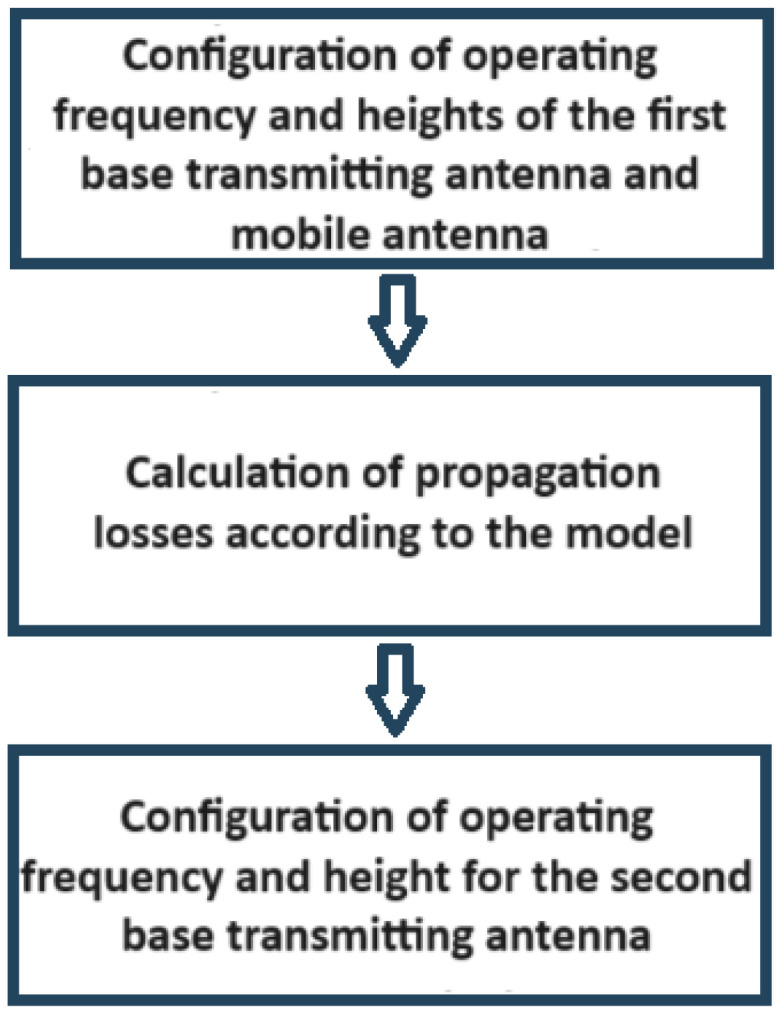
Steps to be repeated in the process to place additional base transmitting antennas.

The web application, thanks to the implemented genetic algorithm, enables the positioning of any number of additional antennas to provide coverage to a wider area in communication services. The process outlined in the schematic diagram of Figure 7 to optimize the placement of two antennas remains unchanged when expanding the number of antennas to be positioned. Only three steps need to be replicated to obtain the optimal coordinates for each new antenna. These additional steps are detailed in the schematic diagram of Figure 10.

To demonstrate the genetic algorithm’s capability to position additional antennas, a 25.31 square kilometer area in Caracas, Venezuela, was selected. This expansive area corresponds to an urban zone with high population density, characterized by numerous tall buildings primarily designated for commerce, businesses, and residences. It also includes hospitals, sports facilities, and wide avenues with sparse vegetation [10].

**Figure 11:**
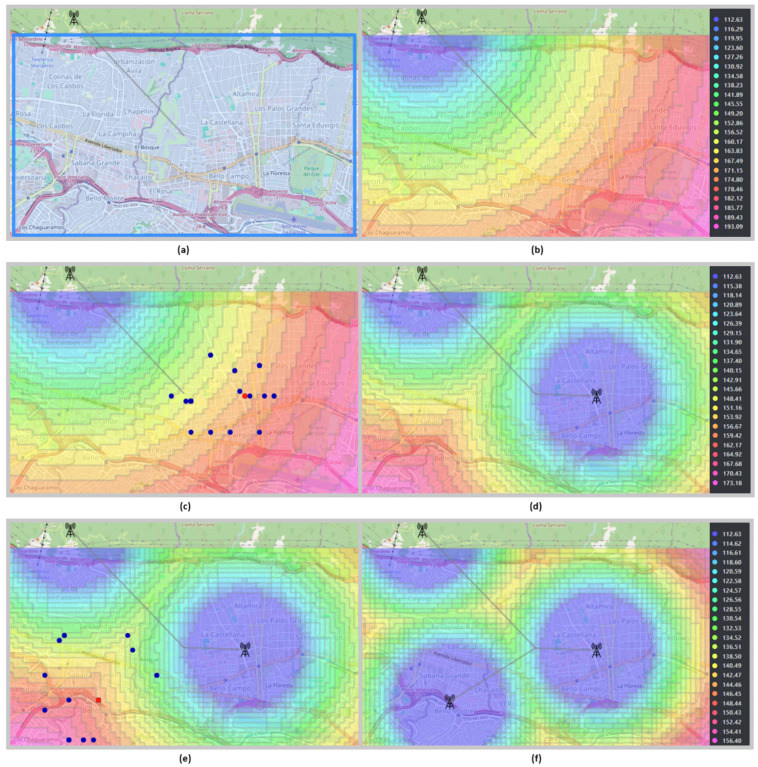
Losses and coverage when placing multiple antennas.

After selecting the area for analysis and following the scheme presented in Figure 7, the installation of the first base transmitting antenna was carried out. In this case, the Ericsson model was chosen to calculate propagation losses. The first transmitting antenna was configured to operate at a frequency of 1500 MHz with a height of 50 m, while the mobile receiver antennas were fixed at a height of 2 m. In Figure 11a,b, the selected area and the associated losses of the first base transmitting antenna are depicted through a color gradient ranging from light blue to pink, with the average losses amounting to 152 dB, a value obtained by applying the function f(x) presented in Section 2.2. Additionally, Figure 11b highlights the coverage provided by the first antenna, visualized in shades of violet.

Once the average losses are calculated, the next step is to configure the second base transmitting antenna for its position. This allows the optimal coordinates for its placement to be determined using the genetic algorithm. In Figure 11c, the optimal point is shown in red where the second base transmitting antenna should be placed, configured in the same way as the first antenna.

Once the second antenna is positioned at the determined optimal coordinates, new propagation losses can be calculated to establish the optimal coordinates for a third base transmitting antenna. Following the process for placing additional antennas, as depicted in Figure 10, the second base transmitting antenna is configured, and propagation losses are then recalculated. The calculation of these new propagation losses involves the two antennas already positioned, as indicated in Section 2.2. Figure 11d displays the updated loss map, illustrating the coverage provided by the two positioned antennas. The new average for propagation losses is identified at 143 dB. Subsequently, the second antenna is reconfigured to determine the optimal coordinates for the third base transmitting antenna, indicated by a red point in Figure 11e. Figure 11f shows the third base transmitting antenna positioned, along with the coverage provided by the three antennas arranged in the selected area. In this scenario, the average for propagation losses decreases to 135 dB, recalling that the calculation of this value involves all the antennas previously positioned.

To position a fourth antenna from the third antenna already positioned, it is necessary to configure the latter, calculate the losses for all three antennas, reconfigure the third antenna, and, thus, determine the coordinates of the fourth transmitting antenna. To optimize the positioning of additional antennas, these steps should be repeated, always considering that a new calculation of propagation losses involves all previously positioned antennas.

As seen in the displayed results, the web application features models for calculating propagation losses and an algorithm for optimizing antenna positioning, enabling analysis of any area to provide coverage for communication services.

### 3.4. Discussion

The introduction of this work provided a comprehensive overview of the advancement of various techniques aimed at optimizing the location of antennas, particularly highlighting the transformative impact of genetic algorithms in this field. Although previous research, such as that mentioned in [23], has explored location optimization methods, [35] pointed out the inherent challenges of applying these techniques in real-world scenarios, thus complicating the comparability of solutions to this complex problem [8,9,11]. Despite these challenges, the unanimous goal in the literature is to achieve an efficient distribution that minimizes the number of antennas required, optimizing their placement to reduce losses and maximize coverage.

However, a limitation observed in many studies is the lack of detail regarding implementation and results under real-world conditions, instead opting for simulations or general descriptions of methods. When scenarios are real, they are limited in space, not to mention under controlled conditions. This aspect contrasts with the approach of the proposed research, presented in [21], which stands out for validating its effectiveness through field tests. This study, which expands on previous research, not only addresses the optimization of antenna positioning but also introduces a versatile and ubiquitous tool. This tool, developed after years of research, is designed to operate globally through the selection of scenarios in the form of a map, incorporating new models to calculate propagation losses and, thus, optimize the location of multiple antennas. The resulting web platform allows real-time evaluation of different models under various scenarios, as shown in the results section.

This work is inspired by numerous studies, but, as already mentioned, it significantly differs in the optimization methods used and, crucially, in the test scenarios applied. For instance, [36] highlights the use of deterministic simulation tools to analyze propagation losses in the millimeter-wave spectrum, focusing on the deployment of 5G networks in dense urban environments. Similarly, [5,6] explored methods based on radar wave propagation and multilateration algorithms, emphasizing precise localization in MIMO radar systems through tests with a stepped-frequency MIMO radar. On the other hand, [4] proposed reinforcement learning for antenna positioning, testing policies in a simulated environment that reflects real propagation conditions. Moreover, [7,15] investigated Bayesian optimization and the use of radio maps for air-to-ground channels, demonstrating the effectiveness of these techniques for antenna positioning in autonomous vehicles and aerial base stations, respectively, offering new insights towards efficient and advanced solutions in antenna optimization.

## 4. Conclusions

The accurate positioning of antennas is essential to ensure effective coverage and optimal service quality in wireless communications, particularly in the context of exponential growth in mobile connectivity and user density. The APP has evolved significantly over time, from theoretical approaches to practical solutions based on advanced algorithms, including genetic algorithms.

This study focused on the development of innovative web tools that leverage genetic algorithms to enhance signal coverage and optimize antenna positioning. Seven different empirical models were explored to calculate propagation losses and were integrated into these antenna positioning tools. The results demonstrate that, with proper configuration and careful model selection, a detailed analysis of antenna positioning in any area is achievable.

For future research, implementing a server capable of analyzing the environment based on the selection of a specific area is suggested, which would enhance the precision and objectivity of the tools in antenna positioning analysis.

## Figures and Tables

**Table 1:** Reviewed propagation models.

Model	Environment Differ	Frequency	Station Height	Mobile Height	Distance
Hata	Yes	150–1500 MHz	30–200 m	1–10 m	1–20 km
COST	Yes	150–2000 MHz	30–200 m	1–10 m	1–20 km
FSPL	No	NOT_LIMITED	NOT_REQUIRED	NOT_REQUIRED	NOT_LIMITED
Egli	No	30–1000 MHz	>1 m	>1 m	1–50 km
ECC 33	Yes	700–3500 MHz	20–200 m	5–10 m	≤10 km
Ericsson	Yes	150–1900 MHz	30–200 m	1–10 m	1–20 km
Sui	Yes	1900–11,000 MHz	10–80 m	2–10 m	0.1–8 km

**Table 2:** Average loss and position optimization for different propagation models.

Model	Environment	Average Area Loss (DB)	Position Optimization (LAT, LON)
Hata	Urban	131	40.509, −3.343
	Suburban	121	40.505, −3.344
	Rural	102	40.508, −3.346
Cost	Urban	133	40.508, −3.342
	Suburban	130	40.507, −3.346
	Rural	126	40.503, −3.344
FSPL	No depend	89	40.507, −3.346
Egli	No depend	108	40.507, −3.348
ECC−33	Urban	118	40.507, −3.345
	Suburban	115	40.508, −3.344
	Rural	115	40.508, −3.344
Ericsson	Urban	125	40.508, −3.344
	Suburban	118	40.506, −3.342
	Rural	107	40.503, −3.342
Sui	Urban	126	40.508, −3.343
	Suburban	122	40.507, −3.344
	Rural	120	40.507, −3.344

## Data Availability

Data are contained within the article.

## Abbreviations

The following abbreviations are used in this manuscript:

BS	Base station
MS	Mobile station
APP	Antenna positioning problem
